# Supplementary material for: Efficacy of acupuncture in the treatment of post-stroke depression: A study protocol of a randomized controlled trial
Source: PLoS One. 2024 Aug 28;19(8):e0303162. doi: 10.1371/journal.pone.0303162 (PMC11357092; doi:10.1371/journal.pone.0303162)
Supplement: S1 Protocol — (PDF) [file pone.0303162.s002.pdf]

Clinical study protocol of acupuncture for post-stroke depression

(Version No.1.2 220230307)

sponsor: First Affiliated Hospital of Tianjin University of Traditional Chinese Medicine

Scheme design: Li Menghan

Clinical study of acupuncture for depression after stroke

1. research topic

Clinical study of acupuncture for depression after stroke.

2. purpose of research

Observe the clinical efficacy of acupuncture on post-stroke depression, provide new ideas for clinical research, and explore the possible mechanism from the perspective of inflammation.

3. Test background

Post-stroke depression (Poststroke depression, PSD) is a complication of stroke depression, loss and depression, which has attracted more attention in recent years. According to statistics, the incidence of PSD in the first year of stroke is about 10%~15%, and the 5-year incidence is 39%~52%[1], The incidence rate in China is about 34.9%, which is higher in women than in men[2]. P S D The main manifestations are emotional apathy, weight change, sleep, sleep disorders, fatigue, a sense of no value and anhedonia[3], Stroke increases the risk of PSD, and PSD is also an adverse factor affecting stroke recovery, often resulting in higher mortality, poorer functional recovery, significant cognitive impairment and lower quality of life[4]. The occurrence of PSD is the result of social psychological factors and biological factors, and its pathophysiological mechanism is complex, mainly related to the regulation of hypothalamic-pituitary-adrenal (HPA) axis, increased inflammatory factors, decreased levels of monoamines, glutamate-mediated excitotoxicity and abnormal neurotrophic response[5-6]. Among them, the inflammatory response theory is one of the focus of PSD mechanism research, a variety of inflammatory factors involved in the occurrence of PSD, mainly by pro-inflammatory cytokines and anti-inflammatory cytokines mutual antagonism, and in different stages of PSD differential expression, through antidepressants can cause changes of inflammatory factors, so change the balance of

inflammatory factors may be one of the mechanisms of treating PSD[7]。

Currently, the first clinical line treatment of PSD is selective serotonin reuptake inhibitors (Selective Serotonin Reuptake Inhibitor, SSRIs), which can prevent and treat depression in PSD patients, but increase adverse reactions such as cerebral hemorrhage, epilepsy, and gastrointestinal symptoms[8]. Considering the limitations of drug therapy, several non-drug therapies have been used for clinical treatment of PSD, including noninvasive brain stimulation, psychotherapy, exercise therapy, acupuncture, music, literary and artistic means[9]. Acupuncture is an important part of traditional Chinese medicine in the treatment of PSD. Many clinical reports show that acupuncture can significantly improve depressive symptoms in patients with PSDLive quality[10-14]. Systematic evaluation showed that acupuncture was significantly effective in improving HAMD and NIHSS scores[15-16], Another study showed that electroacupuncture was no less than antidepressants in improving symptoms in patients with PSD and had a higher safety profile[17]. Acupuncture for the treatment of PSD is the overall regulation of multiple targets, and the specific mechanism mainly includes

Regulating neurotransmitters, improving neuroendocrine disorders, reducing inflammatory factors, reducing oxidative stress, promoting neuronal protection, and promoting regeneration[18]. The author's previous research shows that[19], Acupuncture can reduce the level of proinflammatory factors and increase the level of anti-inflammatory factors to regulate the dynamic balance of inflammatory factors, and may be one of the mechanisms by which acupuncture can improve PSD. In the early small sample of clinical observation found that acupuncture can improve the depression symptoms, improve limb movement function, improve the quality of life. Therefore, while evaluating the clinical efficacy of acupuncture treatment of PSD, observe the effect of acupuncture on inflammatory factors in PSD patients, further explore the mechanism of acupuncture treatment of PSD, to provide a theoretical basis for clinical acupuncture treatment of PSD.

IV. Overall design of the test

1. Overall design type

This study used a single center, randomized controlled design. In patients with post-stroke depression, Both groups underwent conventional acupuncture treatment according to the "stroke" in the textbook "Acupuncture", The control group received oral escitalopram tablets, Trial group plus acupuncture treatment with antidepressant, With the Hamilton Depression Scale (HAMD17), the Depression Self-Assessment Scale (Self-rating depression scale, SDS), The NIHSS Scale, Modified Barthel Index (MBI), And serum IL 1 β, IL 10,5-HT, BDNF as efficacy indicators, Efficiency to calculate HAMD17 score and determine efficacy, The effect of acupuncture on post-stroke depression and its effect on related inflammatory factors were observed.

2. Sample size estimation

According to the results of the previous small sample clinical study, the Hamilton Depression Scale score decreased in the observation group (Δ HAMD) As a result, the test group decreased by 6. 13 ± 1.96 and 4.57, while the control group

decreased by ± 1.87. The superiority test was used, and the formula was calculated according to the sample size

$$2(Z_{\alpha/2}$$ [illegible] $$\beta)^2 \times \sigma^2$$

[illegible] 2

Where α =0.05,1- β =0.9, σ =1.96, δ = (6. 13-4.57) =1.56, with the same rate of 20%, n=42, or 42 patients in each group.

3. Randomization scheme

The 84 patients who met the criteria were included in a 1:1 ratio, independently using the software by the research nurses without the project. The assigned sequence was the acupuncture group and the double number was the control group. The resulting random assignment sequence was placed into sequentially coded, sealed, opaque envelopes, and when the investigator determined their eligibility, the envelopes were opened in order and assigned to the observation or control group. To minimize bias, the allocation sequence cards in the envelope are force-sensitive recording paper and will be qualified by the investigator before opening the envelope

The name of the subject is written on the surface of the envelope.

4. Blind method

The investigators and operators know the group of patients, and the acupuncture treatment of two acupuncture doctors in the acupuncture department, do not participate in the study design, and are not allowed to communicate with the patient about the choice of acupuncture points at the specified time, blindly evaluate the efficacy, and the efficacy evaluator does not participate in the treatment process of patients, that is, the evaluator does not know the specific grouping of patients. Due to the different methods of acupuncture intervention, the patients are not blinded.

5. Na row standard

1. Diagnostic criteria

Standards for stroke diagnosis: formulated according to the Guidelines for the Diagnosis and Treatment of Acute Ischemic Stroke in China 2018 and the Guidelines for the Diagnosis and Treatment of Cerebral Hemorrhage in China 2019;

Criteria for post-stroke depression: refer to the diagnostic criteria for depression episodes in the Diagnostic and Statistical Manual of Mental Disorders compiled by the American Spiritual Association.″Mood disorders characterized by depression or with severe depressive episodes due to stroke can also be manic or mixed features.(DSM -4) ” 。″Depression disorder from other medical conditions″ (DSM-5 ICD-9-CM code 293.83), mainly prominent persistent depressive mood, or significantly less interest or pleasure in all or almost all activities; evidence from medical history, physical examination or laboratory findings is a direct and pathologic result of other physical disorders; this disorder cannot be better explained by other mental disorders; not only instantaneous; clinically significant pain, or leading to social, occupational, or other important damage. The diagnostic criteria for the diagnosis of depressive episodes in the 3rd edition of China Mental Disorders Classification Scheme and Diagnostic Standards were also referred to.

① Symptoms criteria: low mood is mainly, and at least the following 4: a. Loss of interest in daily activities, no pleasure; b. Significant loss of energy, unexplained persistent fatigue; c. Psychomotor retardation or agitation; d. Self-evaluation is too low, or self-blame, or have a sense of guilt, can reach the level of delusion; e. Difficulties in association, or a significant decline in self-conscious thinking ability; f. Repeated thoughts of dying, or suicidal behavior; g insomnia, or early awakening, or excessive sleep; h loss of appetite, or significant weight loss; i significant loss of libido.

② Serious standard: social function damage, causing pain or adverse consequences.

③ Course criteria: symptom criteria and severity criteria have been met for at least 2 weeks.

2. Inclusion criteria

(1) Meet the diagnostic criteria for post-stroke depression;

(2) experience the first stroke, the duration of the disease is 1 to 6 months;

(3) Age: 35~80 years old (including);

(4) The Hamilton Depression Scale (HAMD-17) score of 8-17 points (including);

(5) Clear awareness, stable life signs, and physical examination and cooperation. 3. Exclusion criteria

(1) Those who have received antidepressant drug treatment or are attending other antidepressant clinical trials within 2 weeks;

(2) Patients with consciousness disorder or obvious cognitive impairment (MMSE scale <17 points);

(3) there is a severe aphasia and unable to communicate;

(4) A history of depression before the stroke event occurred;

(5) Patients with severe liver and renal dysfunction (alanine transaminase exceeds 3 times the normal upper limit, and blood creatinine> 180 μ mol / L);

(6) pregnant and lactating women;

(7) Intolerant or refuse to accept acupuncture / electroacupuncture.

4. Removal and shedding standards

(1) If serious adverse events, the trial is stopped according to the doctor's judgment;

(2) The disease progress during the trial, which affects the efficacy and safety determination;

(3) Poor subject compliance (<80% trial treatment compliance), or subjects automatically receiving other treatment; 5. Discontinuation, study criteria

(1) Serious safety problems occurred during the study;

(2) The effect of acupuncture is much lower than that of the western medicine group, so this study does not have clinical value.

Vi. Treatment plan

1. Basic treatment of stroke

For stroke and its high-risk basic diseases (such as hypertension, diabetes, etc.), both groups received symptomatic treatment by referring to the 2019 edition of the Guidelines for the

Clinical Management of Cerebrovascular Diseases in China.

2. Treatment plan

In view of stroke dysfunction, conventional acupuncture was treated according to the "stroke disease" in the textbook "Acupuncture".

Point: ditch, inner pass (both sides), three Yin cross (affected side), spring (affected side), foot (affected side), foot (affected side), committee (affected side), valley (affected side), foot (affected side).

Operation: The patient was placed in the supine position, with 75% alcohol and skin disinfection, and the disposable acupuncture needle (Huatuo brand, Suzhou Medical Supplies Factory Co., LTD., specification: 0.25mm 40mm) was used for acupuncture. The ditch is 5-10 mm to the nasal septum, peck to wet the eye; 10-15 mm, and thrust for 1 min; the affected limb is smoked 3 times, the depth of the other acupoints is 20-25 mm and the needle is left for 30 minutes. Acupuncture was performed 6 times weekly for 4 weeks of treatment. (1) Control group: oral escitalopram tablets for 10mg Q d daily for 4 weeks. (2) Test group: increase antidepressant acupuncture treatment.

Take point: hundred hui, Yin tang, wind house, eararmor.

Operation: The patient took the semi-decubitus position, and the bed was raised 45°. After 75% alcohol disinfection, the "brain opening" acupuncture was performed first. The method was the same as before, and then the anti-depression related acupoints were acupuncture after getting gas. Baihui flat stab 3~5mm, Yintang to nasal root 10~15mm, wind needle to 15~20mm, eararmor is equivalent to 2~ 3mm in the heart and kidney, then a group of electric acupuncture (SDZ-IIB electric needle instrument), frequency is 2 / 15 Hz, strength 2 mA, time 30min, two sides of the ear point alternating acupuncture / electric acupuncture. Acupuncture was performed 6 times weekly for 4 weeks of treatment. 1. Observation indicators 1.1 Demographic data: age, gender, height, weight, educational level, course of disease, concomitant drug use, etc.1.2 General physical examination: body temperature, heart rate, respiration, blood pressure, etc.2. The effect index 2.1 main efficacy index Hamilton depression scale (HAMD17): used to assess the severity of depression symptoms, depression depression including depression, guilt, suicide, sleep difficulty, sleep, early, work and interest, slow, provoke, mental anxiety, physical anxiety 17 items, is the most widely used.2.2 Secondary efficacy indicators

① Clinical effectiveness: the efficacy index was calculated by the reduced value of HAMD17 score and the efficacy was determined.②

Depression Self-assessment Scale (Self-rating depression scale, SDS): It is used for the self-assessment of post-stroke depression patients, including 20 items, and is commonly used clinically.

③NIHSS: To assess the severity of neurological deficit symptoms in stroke patients.

④ Modified Barthel Index (MBI): used to evaluate the self-care ability of stroke patients, the higher the score, the stronger the self-care ability.

⑤ Laboratory indicators: serum IL 1 β, IL 10,5-HT, BDN F levels.

⑥ Serum metabolomic analysis. Methods: After thawing at room temperature, 100 μ L was transferred to a new tube, adding 300 μ L methanol and 10 μ L internal standard (2-chlorophalanine 2.5 g / L) to each sample. After the samples were mixed and centrifuged, 200 μ L of supernatant was absorbed into the sample tube for liquid chromatography MS testing using the Acquity TM UPLC-Q-TOF-MS platform (Al fa Chemistry, USA). The extraction of the advanced data features of the obtained data, after the XCMS processing in R, was normalized and formed into a two-dimensional data matrix.

2.3 Efficacy determination criteria

The efficacy index was calculated from the Hamilton Depression Scale (HAMD17) as [(total pre-therapy-total post-therapy) / total pre-therapy] 100%. The efficacy was determined as follows:

Recovery: a 75% reduction in syndrome points;

Significant effect: 50% syndrome score reduction <75%;

Effective: 25% syndrome score reduction <50%;

Invalid: <25%.

Total response rate = (recovery + significant + effective) / 100% total cases

3. Safety indicators

Possible adverse reactions and incidence of adverse reactions.

4. Observation time point

The scales were evaluated before, 2 weeks and 4 weeks of intervention, and serum indicators as well as metabolomics analysis were tested before and after 4 weeks of treatment.

5. Statistical analysis

All the data were analyzed by SPSS 26.0 meter software. The measurement data were tested for normality and homogeneity of variance first. For those conforming to the normal distribution, independent sample t-test was used, and for those not conforming to

the normal distribution, non-parametric test was used. Count data were tested by chi-square (χ^2). The grade data are tested as non-parametric tests. A $P < 0.05$ was considered as a statistically significant difference.

6. Record and reporting of adverse events

6.1 Record of adverse events

In the case report form, the "Adverse Event Record Form" is set up to require the investigator to truthfully fill in the occurrence time, severity, duration, measures taken and outcome of the adverse event.

.26 Adverse event reporting

If any serious adverse event occurs in the trial, the investigator must take immediate measures to protect the safety of the subjects, and timely report to the research responsible unit and the ethics committee. The investigator should sign and date the report. The Sponsor will guarantee reporting procedures meeting all legal and regulatory requirements.

6.3 Handling of serious Adverse events

If serious adverse events occur in the trial, such as cerebrovascular accident, myocardial infarction, the test should be stopped immediately and treated symptomatic; if the patient is receiving acupuncture treatment for the first time, the doctor should explain to prevent acupuncture sickness. If case, we should refer to the acupuncture accident in the "acupuncture moxibustion Law" (planning textbook), immediately stop treatment, let the patient take his head and low position, rest for a while, and give warm boiled water; on the basis of the above treatment, can moxibustion Baihui, air sea, Guan Yuan, Shenque; if not relieved, should give emergency treatment. If injection occurs, apply hematoma immediately for bleeding for 1 minute, and ask the patient to apply cold compress on the same day and heat compress the next day; if local infection occurs, anti-inflammatory treatment should be given.

8. Number, according to the management

All people involved with data collection need to be confirmed and describe their work. Clinical investigators must fill in the CRF accurately, timely, completely and properly according to the original data information. CRF, the modification of the data must follow the standard operating procedures and retain the modification traces. Once errors or discrepancies are identified, the investigator should

be notified to ensure all data recorded and reported are correct and complete. If necessary, the data management plan can be updated and revised in time according to the changes in the study protocol, but the corresponding procedures need to be completed. Data managers shall conduct adequate verification of the primary and secondary effectiveness indicators and key safety indicators specified in the protocol to ensure the correctness and completeness of these data.

IX. Quality control

Implement the process quality control and data quality control. Including investigator compliance control, subject compliance control, and follow-up information integrity control. Data quality control includes data supervisor personnel composition, responsibilities and workflow, entry process, mid-term analysis plan, study termination standard, data verification, data cleaning, etc.

X. Research ethics requirements and statements

This clinical study will follow the Declaration of Helsinki (2010 edition) and relevant Chinese clinical research practices

Stattes proceed. The study shall be conducted only before the approval of the ethics committee of the responsible unit. Before each patient is enrolled for the study, the study physician has the responsibility to provide a complete, comprehensive presentation of the purpose, procedures or the possible risks of the study. Patients should be informed that they have the right to withdraw at any time. An informed consent form must be given to each patient before inclusion. It is the responsibility of the study physician to ensure that each patient signs the informed consent form before entering the study and remains in the study file.

For patients with possible pregnancy, the patient must be informed that if they are pregnant during the study, the study may pose a risk to the fetus, and the patient must use contraception to participate in the study. If the patient is suspected to be unable to do so, they must not be admitted to the study.

Since the study involves a clinical trial, the investigator declares that the results will be published whether the relevant study results are positive or negative.

References: References:

[1] Towfighi A , Ovbiagele B , El Husseini N , et al .Poststroke Depression : A Scientific Statement for Healthcare Professionals From the American Heart Association /American Stroke Association [J].Stroke .2017;48(2):e30-e43.

[2] Li Shiming, Feng Wei, Cui Fengwei, et al. A meta-analysis of the prevalence of depression after stroke in China [J]. Journal of Practical Medicine, 2021,37 (16): 2058-2064.

[3] Feng C , Fang M , Liu XY .The neurobiological pathogenesis of poststroke depression [J].TheScientificWorldJournal .2014:521349.

[4] Cai W , Stewart R , Mueller C , et al .Poststroke depression and risk of stroke recurrence and mortality : protocol of a meta-analysis and systematic review[J].BMJ open.2018;8(12):e026316. [5] Guo J , Wang J , Sun W , et al .The advances of post -stroke depression : 2021 update [J].Journal of neurology.2022;269(3):1236-49.

[6] Qi Shikui, Gao Jing, Yu Mingyue, and etc. Progress in the pathogenesis of depression after stroke [J]. Stroke and Neurological Diseases, 2022,29 (05): 483-486.

[7] Tang Wenjing, Wu Siyuan, Yang Chen, et al. Inflammatory response and poststroke depression [J]. China Tissue Engineering Research, 2022,26 (08): 12781285.

[8] Kalbouneh HM , Toubasi AA , Albustanji FH , et al .Safety and Efficacy of SSRIs in Improving Poststroke Recovery : A Systematic Review and Meta -Analysis [J].Journal of the American Heart Association .2022;11(13):e025868.

[9] Wijeratne T , Sales C , Wijeratne C .A Narrative Review on the Non - Pharmacologic Interventions in Post -Stroke Depression [J].Psychology research and

behavior management .2022;15:1689-706.
[10] You Y , Zhang T , Shu S , et al .Wrist -ankle acupuncture and Fluoxetine in the treatment of post -stroke depression : a randomized controlled clinical trial [J].Journal of traditional Chinese medicine .2020;40(3):455-60.
[11] Yin XJ , Wang F , Lin GP , et al .Effects of auricular acupressure on depression in stroke patients : A single -blind randomized controlled trial [J].Complementary therapies in clinical practice .2022;48:101596.
[12] Li MH , Zhang B , Meng ZH , et al .Effect of Tiaoshen Kaiqiao acupuncture in the treatment of

ischemic post-stroke depression: a randomized controlled trial.Journal of traditional Chinese medicine [J].2017;37(2):171-8.
[13] Cai W, Ma W, Li YJ, et al.Efficacy and safety of electroacupuncture for post-stroke depression: a randomized controlled trial [J].Acupuncture in medicine : journal of the British Medical Acupuncture Society.2022;40(5):434-42.
[14] Huang Haipeng, Yu Bin, Guan Qifan, etc. Progress in clinical study of acupuncture for depression after stroke [J]. Chinese Journal of Gerontology, 2022,42 (18): 4617-4621.
[15] Zhang L, Chen B, Yao Q,et al.Comparison between acupuncture and antidepressant therapy for the treatment of poststroke depression: Systematic review and meta-analysis [J].Medicine.2021;100(22):e25950.
[16] Hang X, Li J, Zhang Y, et al.Efficacy of frequently-used acupuncture methods for specific parts and conventional pharmaceutical interventions in treating post-stroke depression patients: A network meta-analysis [J].Complementary therapies in clinical practice.2021;45:101471.
[17] Wang XF, Cai W, Wang YP,et al.Is Electroacupuncture an Effective and Safe Treatment for Poststroke Depression?An Updated Systematic Review and Meta-Analysis [J].BioMed research international.2021;2021:8661162.
[18] CAI Wa, Gao Hang, Wei Xifang, et al. Progress in the mechanism of acupuncture for post-stroke depression [J]. Traditional Chinese Medicine Guide, 2021,27 (09): 164167.
[19] Li M, Ding R, Yang X, Ran D.Study on Biomarkers Related to the Treatment of Post-Stroke Depression and Alternative Medical Treatment Methods.Neuropsychiatr Dis Treat.2022 Aug 26;18:18611873.

Xi. Schedule

1. The Hamilton Depression Scale (HAMD-17)

The Hamilton Depression Scale (HAMD-17) (please type "×") in the "□" before the answer closest to the patient's true situation	
1 Depression mood	□0Symptomless<br>□1The complaint is stated only when asked<br>□2Be pressed spontaneously in speech<br>□3This emotion can be expressed from expression, posture, voice, or crying without words<br>□4The patient's spontaneous and non-spontaneous language (expressions, movements) are almost completely manifested by this emotion
2 Guilty	□0Symptomless<br>□1Blame yourself and feel that you have affected others<br>□2Think that you have committed a crime, or think twice about past mistakes and mistakes<br>□3That the current disease is a punishment for their own mistakes, or a criminal delusion<br>□4Criminal delusion is accompanied by accusations or threatening hallucinations
	□0Symptomless<br>□1There's no point in living

3 Suicide	☐**2**I hope I have died, or I often think of something related to death<br>☐**3**Negative perception (suicidal thoughts)<br>☐**4**Serious suicidal behavior
4. Difficulty in falling asleep	☐**0**Symptomless<br>☐**1**The chief complaint sometimes has difficulty falling asleep, that is, half an hour after going to bed

	☐**2**The chief complaint had difficulty falling asleep every night
5 Sleep is not deep	☐**0**Symptomless<br>☐**1**Sleep shallow many evil dreams<br>☐**2**Wake up in the middle of the night (before 12 pm) (excluding bathroom use)
6 Wake up early	☐**0**Symptomless<br>☐**1**Wake up early, an hour earlier than usual, but you can fall asleep again<br>☐**2**I can't fall asleep again after waking up early
7. Work and interests	☐**0**Symptomless<br>☐**1**Ask questions and tell them<br>☐**2**Spontaneously directly or indirectly express a loss of interest in activities, work or study, such as feeling listless, Indecision, unable to insist or need to force themselves to work or activity<br>☐**3**Hospital labor or entertainment for less than 3 hours<br>☐**4**Stop working due to the current illness, hospitalized patients do not participate in any activities or cannot complete the daily affairs without the help of others
8 Slow: refers to slow thinking and language, difficulty in concentration, and loss of initiative.	☐**0**Symptomless<br>☐**1**Mild delay was noted on the psychiatric examination<br>☐**2**Significant mental retardation was found on the mental examination<br>☐**3**Mental examination is difficult to conduct<br>☐**4**Unable to answer questions (stiff)
9 The more exciting	☐**0**Symptomless<br>☐**1**Check the examination<br>☐**2**Obviously distracted or small movements<br>☐**3**Can not sit still, ever standing during the examination<br>☐**4**Rub your hands, bite your fingers, pull your hair, and bite your lips
10 Mental anxiety	☐**0**Symptomless<br>☐**1**When asked, he complained<br>☐**2**Spontaneous expression<br>☐**3**The expression and speech reveal obvious anxiety<br>☐**4**Apparently frightened
11. Somatic anxiety:<br>It refers to the physiology of anxiety, and its symptoms, including dry mouth, abdominal distension, diarrhea, beating, abdominal cramps, palpitations, headache, excessive ventilation and sighing, and frequent urination and	☐**0**Symptomless<br>☐**1**mild<br>☐**2**Moderate, with positive above symptoms<br>☐**3**Severe, the above symptoms are serious, affect the life or need to be treated<br>☐**4**Seriously affect life and activities

sweating	
12 Gastrointestinal symptoms	☐**0**Symptomless<br>☐**1**Loss of appetite, but eat by yourself without any encouragement<br>☐**2**Eating requires urging or requests or laxatives or digestive aids
13 Systemic symptoms	☐**0**Symptomless<br>☐**1**Limbs, back, or neck heaviness, backache, headache, muscle pain, general fatigue or fatigue ☐**2**The above symptoms are obvious
14. Sexual symptoms: Refers to decreased sexual desire, menstrual disorders, etc	☐**0**Symptomless<br>☐**1**mild<br>☐**2**severe;

	Can not be sure, or this item is not suitable for the evaluated candidate.(Excluding the total score)
15 Suspected disease	□**0**Symptomless<br>□**1**Too much attention to the body<br>□**2**Think repeatedly consider health issues<br>□**3**Suspicion<br>□**4**A suspected disease delusion with hallucinations
16 Weight loss	□**0**Symptomless<br>□**1**Weight loss of more than 1 kg within a week<br>□**2**Weight loss of more than 2 kg within a week
17 Self-knowledge	□**0**Know oneself sick, the performance is melancholy<br>□**1**Know you are sick, but due to poor food, environmental problems, busy work, virus infection or need to rest<br>□**2**Completely deny illness
Note: The code indicates that the score is 0=0 points, 1=1 points, 2=2 points, 3=3 points, 4=4 points	

2. Depression Self-Assessment Scale (SDS)

Depression Self-Assessment Scale (SDS)<br>Description: According to the actual situation of the subject, type "" in the "□" before the answer closest to the actual situation of the patient.	
	code of points
1 I felt unhappy and depressed.	□**1**a fat lot□**2**A small part of the time□**3**A considerable amount of time□**4**Most of the time
2 I think the morning is the best in all day.	□**4**a fat lot□**3**A small part of the time□**2**A considerable amount of time□**1**Most of the time
3 I burst out crying or felt like I wanted to cry.	□**1**a fat lot□**2**A small part of the time□**3**A considerable amount of time□**4**Most of the time
4. I don't sleep very well at night.	□**1**a fat lot□**2**A small part of the time□**3**A considerable amount of time□**4**Most of the time
5 I eat as much as usual.	□**4**a fat lot□**3**A small part of the time□**2**A considerable amount of time□**1**Most of the time
6 I was as happy as ever in close contact with the opposite sex.	□**4**a fat lot□**3**A small part of the time□**2**A considerable amount of time□**1**Most of the time
7 I noticed that my weight was losing weight.	□**1**a fat lot□**2**A small part of the time□**3**A considerable amount of time□**4**Most of the time
8 I have constipation.	□**1**a fat lot□**2**A small part of the time□**3**A considerable amount of time□**4**Most of the time
9 My heart beats faster than usual.	□**1**a fat lot□**2**A small part of the time□**3**A considerable amount of time□**4**Most of the time
10 I feel tired for no reason.	□**1**a fat lot□**2**A small part of the time□**3**A considerable amount of time□**4**Most of the time
11 My mind is as clear as usual.	□**4**a fat lot□**3**A small part of the time□**2**A considerable amount of time□**1**Most of the time
12 I don't think doing things is difficult.	□**4**a fat lot□**3**A small part of the time□**2**A considerable amount of time□**1**Most of the time

13 I felt uneasy and restless.	□1a fat lot□2A small part of the time□3A considerable amount of time□4Most of the time
14 I have hope for the future.	□4a fat lot□3A small part of the time□2A considerable amount of time□1Most of the time
15 I’m more angry than usual.	□1a fat lot□2A small part of the time□3A considerable amount of time□4Most of the time
16 I think it is easy to make a decision.	□4a fat lot□3A small part of the time□2A considerable amount of time□1Most of the time
17 I feel like a useful person, and someone needs me.	□4a fat lot□3A small part of the time□2A considerable amount of time□1Most of the time
18 I have had a very interesting life.	□4a fat lot□3A small part of the time□2A considerable amount of time□1Most of the time
19 I think other people will live better if I die.	□1a fat lot□2A small part of the time□3A considerable amount of time□4Most of the time
20 I am still interested in what I am usually interested in.	□4a fat lot□3A small part of the time□2A considerable amount of time□1Most of the time

grade:	__	__	component

The 3.NIHSS scale

National Center of Health Neurological Deficiency Score (NIHSS)

Fill in the form description: Please read each item carefully, clearly understand each item, conduct clinical physical examination, and then score according to the actual situation of the patient.(Fill in by the clinician), please type "" in the "☐" before the answer closest to the true situation of the patient.

number	check up	code of points	score
1a	level of consciousness:<br>Even if there is no comprehensive evaluation (such as endotracheal intubation, language impairment, tracheal trauma, bandage dressing, etc.), the examiner must choose one reaction.3 points only if the patient does not respond to noxious stimuli (not reflex).	0= sober, and responsive<br>1= drowsiness, the minimum stimulation that awakens the patient to complete instructions, answer questions, or respond<br>2= drowsy or unresponsive, requiring intense repeated or painful stimulation to have a non-fixed pattern response<br>3= only reflex activity or spontaneous response, or complete no response, soft paralysis, no response	☐**0**=0 points for<br>□**1**=1 point<br>□**2**=2 points for<br>□**3**=3 Points
1b	Level of consciousness question:<br>Month, age. Rcore only for initial responses. Mark 2 points for aphasia and unconscious people who do not understand the problem, and 1 point for endotracheal intubation, tracheal trauma, severe dysarthria, language impairment or any other reason (not caused by aphasia). A written answer is available.	0= both terms are correct<br>1= A correct<br>2= both items are incorrect	☐**0**=0 points for<br>□**1**=1 point<br>□**2**=2 Points
1c	Level of awareness instruction:<br>Open eyes; unparalyzed side fist grip release. For the initial response score only, those with clear effort but without completion were also scored. If there is no response to the instruction, use the action and record the score. Appropriate instructions should be given to those with trauma, amputation, or other physiological defects.	0= both terms are correct<br>1= A correct<br>2= both items are incorrect	☐**0**=0 points for<br>□**1**=1 point<br>□**2**=2 Points
2	gaze fixedly:<br>Only the horizontal eye movements were tested	0= Normal<br>1= partial gaze palsy (monocular or binocular gaze abnormalities, but no passive or complete gaze palsy)<br>2= passive gaze or complete gaze paralysis (can not be	☐**0**=0 points for<br>□**1**=1 point<br>□**2**=2

		overcome by eye-head movements)	Points
3	visual field:<br>The lower and upper quadrants were detected by hand index or visual threat method	0= no visual field is missing<br>1= Part of the blindness<br>2= all, all biased blindness<br>3= bilateral hemianopia (total blindness, including cortical blindness)	☐**0**=0 points for<br>□**1**=1 point<br>□**2**=2 points for<br>□**3**=3 Points
4	facioplegia	0= normal 1= minimal (nasolabial groove flattening, asymmetric when smiling)<br>2= Part (complete or almost complete paralysis of the lower parts, central paralysis)<br>3= complete (single or bilateral paralysis, lack of upper and lower facial movement, peripheral paralysis)	☐**0**=0 points for<br>□**1**=1 point<br>□**2**=2 points for<br>□**3**=3 Points
5	upper extremity exercise:<br>Put the limb in the appropriate position: sit when the upper limb flat lift 90°, Lift 45 while lying on your back^{o}, Palmar down, if the upper limb in 10 seconds, remember 1~4 points.<br>Encourage the aphasia with language or action, without harmful stimulation. Each limb was examined in turn, starting from the upper limb on the nonparalyzed side.	Upper limb: 0= upper limb in the required position for 10 seconds, no fall<br>1= The upper limb can be lifted, but not for 10 seconds, do not hit the bed or other support when falling	☐**0**=0 points for<br>□**1**=1 point<br>□**2**=2 points for<br>□**3**=3 points for<br>□**4**=4 Points

		2= against some gravity but the upper limb cannot reach or maintain the sitting 90 ° or recumbent position 45° , faster fall to bed<br>3= no antigravity, faster fall of upper limb 4= no movement 9= amputation or joint fusion, explanation: 5a left upper limb 5b right upper limb	☐**9**=9 Points
6	Lower limb movements:<br>Place the limb in the appropriate position: raise the lower limb in the horizontal position of 30°, If the lower limbs fall within 5 seconds, record 1 to 4 points. Encourage the aphasia with language or action, without harmful stimulation. Each limb was examined in turn, starting from the upper limb on the nonparalyzed side.	lower limbs:<br>0= stick to the required position for 5 seconds, not falling 1= fall at the end of 5 seconds, do not hit the bed 2 = fall to the bed within 5 seconds, but can resist gravity<br>3= fast fall, unable to resist gravity<br>4= No movement<br>9= Amputation or joint fusion, interpretation: 6a Left lower limb 6b right lower limb	☐**0**=0 points for □**1**=1 point □**2**=2 points for □**3**=3 points for □**4**=4 points for □**9**=9 Points
7	incoordination:<br>Bilateral digital nose and knee tibial tests, scored when ataxia and weakness are not proportional. If the patient can not understand or limb paralysis is not scored.	0= no ataxia 1= one limb with 2= two limbs with, ataxia in:<br>Right upper limb 1= yes, 2= none<br>9= Amputation or joint fusion, interpretation:<br>Left upper limb 1= yes, 2= none<br>9= Amputation or joint fusion, interpretation:<br>Right upper limb 1= yes, 2= none<br>9= Amputation or joint fusion, interpretation:<br>Left lower extremity 1= yes, 2= none<br>9= Amputation or joint fusion, interpretation:<br>Right lower extremity 1= yes, 2= none	☐**0**=0 points for □**1**=1 point □**2**=2 points for □**9**=9 Points
8	sense perception:<br>A drowsy or aphasia can score 1 or 0. Brain stem stroke in bilateral sensory loss, no response and tetraplegic patients, coma patients recorded 2 points.	0= Normal<br>1= mild to moderate, the affected side of the needle prick sensation is not obvious or blunt or only tactile<br>2= severe to complete sensory loss, no face, upper and	☐**0**=0 points for □**1**=1 point □**2**=2

		lower limbs	Points
9	language:<br>Naming, reading the test. Coma patient with 3 points.	0= Normal, with no aphasia<br>1= Light to moderate: some defects in fluency and comprehension, but no significant limitation in expression.<br>2= serious aphasia, communication is through the patient's broken language expression, the listener must reason, ask, guess, can exchange the scope of information is limited, the examiner feels communication difficulties.<br>3= dumb or complete aphasia, unable to speak or understand	□**0**=0 points for □**1**=1 point □**2**=2 points for □**3**=3 Points
10	dysarthrosis:<br>If the patient is unable to speak for endotracheal intubation or other physical obstacles, score 9 points.	0= Normal<br>1= Light to moderate, at least some unclear pronunciation, although difficult, but can be understood<br>2= unclear speech and cannot be understood 9= endotracheal intubation or other physical disorder, explanation:	□**0**=0 points for □**1**=1 point □**2**=2 points for □**9**=9 Points
11		0= no neglect disorder	□**0**=0 Points

	Neglect:<br>If the patient is aphasia, but does show bilateral attention, the score is normal.	1= sight, touch, hearing, spatial perception, or personal neglect; or loss of bilateral simultaneous stimuli to either sensation<br>2= serious partial neglect; more than one form of partial neglect; not knowing your hand, only one side space	□1=1 point<br>□2=2 Points
Note: The code represents the score.		grade: ____ component	

4. Modified Barthel Index (MBI)

Modified Barthel Index (MBI) (please select one of the alternative results and mark the "×") in the corresponding "□"	
Daily activity items	code of points
1 Eat	□10Completely independent□8A small amount of help□5Medium help 2 and lots of help□□0be completely dependent on
2 Take a bath	□5Completely independent□4Small help 3 medium help 1 large help□□□0be completely dependent on
3 Personal hygiene	□5Completely independent□4Small help 3 medium help 1 large help□□□0be completely dependent on
4 Dress (including tying shoelaces)	□10Completely independent□8A small amount of help□5Medium help 2 and lots of help□□0be completely dependent on
5 Fecal control	□10Completely independent□8A small amount of help□5Medium help 2 and lots of help□□0be completely dependent on
6 Urine control	□10Completely independent□8A small amount of help□5Medium help 2 and lots of help□□0be completely dependent on
7 Go to the toilet (including cleaning and arranging clothes after using the toilet)	□10Completely independent□8A small amount of help□5Medium help 2 and lots of help□□0be completely dependent on
8 The bed chair transfer	□15Completely independent□12A small amount of help□8Medium help 3 massive help□□0be completely dependent on
9 Flat ground walking (45m) / wheelchair operation	□15Completely independent□12A small amount of help□8Medium help 3 massive help□□0be completely dependent on
10 to take up and down the stairs	□10Completely independent□8A small amount of help□5Medium help 2 and lots of help□□0be completely dependent on
Note: The code indicates that the score is 15=15 points 12=12 points 10=10 points 8=8 points 5=5 points 2=2 points 1=1 point<br>0=0 Points	

针刺治疗脑卒中后抑郁的临床研究方案

（版本号 **1.2**　版本日期 **20230307**）

申办单位：　天津中医药大学第一附属医院

方案设计：　李孟汉

针刺治疗脑卒中后抑郁的临床研究

一、研究题目

针刺治疗脑卒中后抑郁的临床研究。

二、研究目的

观察针刺治疗脑卒中后抑郁的临床疗效，为临床研究提供新思路，从炎症角度探讨可能的机制。

三、试验背景

卒中后抑郁（Poststroke depression，PSD）是脑卒中事件发生后的以兴趣丧失、情绪低落为主要表现的情感障碍并发症，近年来已引起较多的关注。据统计PSD 在脑卒中第一年发病率约为 10%～15%，5 年发病率为 39%～52%[1]，我国发病率约为 34.9%，女性高于男性[2]。PSD 主要表现为情绪冷漠、体重改变、睡眠障碍、疲劳、无价值感和快感缺乏[3]，脑卒中增加 PSD 风险，而 PSD 也是影响卒中恢复的不良因素因素，常造成较高的死亡率、较差的功能恢复、明显的认知障碍和更低的生活质量[4]。PSD 发生是社会心理学因素和生物学因素综合总用的的结果，其病理生理学机制较为复杂，主要与下丘脑-垂体-肾上腺(HPA)轴的失调、炎症因子的增加、单胺水平的降低、谷氨酸介导的兴奋性毒性和异常的神经营养反应有关[5-6]。其中，炎症反应学说是当前 PSD 机制研究的热点之一，多种炎症因子参与了 PSD 的发生，主要由促炎性细胞因子和抗炎性细胞因子进行相互拮抗，并在 PSD 不同阶段有差异性的表达，通过抗抑郁药物可引起炎症因子的改变，因此改变炎症因子的平衡可能是治疗 PSD 的机制之一[7]。

目前 PSD 临床一线的治疗是选择性 5-羟色胺再摄取抑制剂(Selective Serotonin Reuptake Inhibitor, SSRIs)，可以预防和治疗 PSD 患者的抑郁情绪，但增加了脑出血、癫痫、胃肠道症状等不良反应[8]。考虑到药物治疗的限制，目前已有多种非药物疗法用于临床治疗 PSD，包括无创性脑刺激、心理疗法、运动疗法、针灸、音乐、文学和艺术手段等[9]。针灸是中医学的重要组成部分，治疗 PSD 疗效确切，较多的临床报道表明针刺可显著改善 PSD 患者的抑郁症状，改善生活质量[10-14]。系统评价显示，针灸在改善 HAMD、NIHSS 评分方面效果显著[15-16]，另一项研究表明电针在改善 PSD 患者症状方面不亚于抗抑郁药物，并有较高的安全性[17]。针刺治疗 PSD 是多方面多靶点进行的整体调节，具体机制主要包括

调节神经递质、改善神经内分泌紊乱、降低炎症因子、减少氧化应激、神经元保护及促进再生等方面[18]。笔者前期研究表明[19]，针刺可以减少促炎因子水平，增加抗炎因子水平以调节炎症因子的动态平衡，可能是针刺改善 PSD 的机制之一。在前期小样本的临床观察发现，针刺可改善 PSD 患者的抑郁症状，提高肢体运动功能，改善生活质量，因此通过本项目评估针刺治疗 PSD 临床疗效的同时，观察针刺对 PSD 患者的炎症因子的影响，进一步探讨针刺治疗 PSD 的作用机制，为临床针刺治疗 PSD 提供理论依据具有重要意义。

四、试验总体设计

1.总体设计类型

本研究采用单中心、随机对照设计。以卒中后抑郁患者为研究对象，两组均按照《针灸学》教材中“中风病”针刺方法进行常规针刺治疗，对照组口服艾司西酞普兰片，试验组加用抗抑郁的针刺治疗，以汉密尔顿抑郁量表（HAMD-17）、抑郁自评量表（Self-rating depression scale，SDS），NIHSS 量表，改良 Barthel 指数(MBI)，以及血清 IL-1β、IL-10、5-HT、BDNF 作为疗效指标，以 HAMD-17 减分值计算有效率并进行疗效判定，观察针刺治疗卒中后抑郁的疗效及对相关炎症因子的影响。

2.样本量估算

根据前期小样本临床研究中结果，观察组汉密尔顿抑郁量表评分降低（ΔHAMD）结果，试验组降低 6.13±1.96，对照组降低 4.57±1.87，采用优效性检验，根据样本量计算公式

$$n = \frac{2(Z_{\alpha/2} + Z_{\beta})^2 \times \sigma^2}{\delta^2}$$

其中 α=0.05，1-β=0.9，σ=1.96，δ=(6.13-4.57)=1.56，代入公式并按照 20%的脱失率，可计算出 n=42，即每组需要 42 例患者。

3.随机方案

纳入符合标准的 84 例患者按照 1:1 的比例，由不参与项目的研究护士独立使用软件产生随机分配序列，分配序列单数为针刺组，双数为对照组。产生的随机分配序列被放入按顺序编码、密封不透光的信封，当研究人员确定受试对象的合格性后，按顺序拆开信封并将受试对象分配入观察组或者对照组。为最大限度减少偏倚，信封内的分配序列卡片为力敏型记录纸，研究者在拆开信封前将合格

受试对象的姓名写在信封表面。

4.盲法

研究者及操作者均知道患者所处的组别，全部病例的针刺治疗由针灸科的两名针灸医师负责，针灸医师只需要按照既定的方案进行操作，不参与研究设计，不允许与患者交流关于针刺穴位选择的问题；按规定时间接受针刺治疗，在疗效评价时采用盲法评价，疗效评估者也不参与研究设计，不参加患者的治疗过程,即评价者从而不了解患者的具体分组。因针刺干预方法不同，故不对患者施盲。

五、纳排标准

1.诊断标准

脑卒中诊段标准：参照《中国急性缺血性脑卒中诊治指南 2018》及《中国脑出血诊治指南 2019》制定；

卒中后抑郁诊段标准：参照美国精神协会编制的《精神疾病诊断与统计手册》第 4 版和第 5 版中抑郁发作诊断标准。“由于卒中导致的以抑郁为特征或伴有重型抑郁样发作的心境障碍，亦可表现为躁狂或混合样特征。（DSM-4）”。“其他医学情况所致的抑郁障碍”（DSM-5 ICD-9-CM 编码 293.83），主要临床表现为突出的持续性的抑郁心境，或对所有或几乎所有活动的兴趣或乐趣明显减少；从病史、躯体检查或者实验室发现的证据表明，该障碍是其他躯体疾病的直接的病理生理性结果；这种障碍不能用其他精神障碍来更好的解释；并非仅仅出现于谵妄时；有临床意义的痛苦，或导致社交、职业或其他重要方面的损害。并参考《中国精神疾病分类方案与诊断标准》第 3 版中抑郁发作的诊断标准。

①症状标准：心境低落为主，并至少有下列 4 项：a.对日常活动丧失兴趣，无愉快感; b.精力明显减退，无原因的持续疲乏感；c.精神运动性迟滞或激越；d.自我评价过低，或自责，或有内疚感，可达妄想程度；e.联想困难，或自觉思考能力显著下降；f.反复出现想死的念头，或有自杀行为；g 失眠，或早醒，或睡眠过多；h 食欲不振，或体重明显减轻；i 性欲明显减退。

②严重标准：社会功能受损，给本人造成痛苦或不良后果。

③病程标准：符合症状标准和严重标准至少已持续 2 周。

2.纳入标准

（1）符合卒中后抑郁的诊断标准；

（2）经历首次卒中发病，病程 1～6 月；

（3）年龄 35～80 岁（包括）；

（4）汉密尔顿抑郁量表（HAMD-17）评分 8～17 分（包括）；

（5）意识清楚，生命征平稳，查体合作。

3.排除标准

（1）2 周内接受过抗抑郁药物治疗或正参加其他抗抑郁临床试验者；

（2）存在意识障碍或明显的认知障碍功能（MMSE 量表＜17 分）者；

（3）存在重度失语无法沟通者；

（4）本次卒中事件发生前已有抑郁症病史；

（5）合并严重肝肾功能障碍（谷丙转氨酶超过正常上限 3 倍、血肌酐＞180μmol/L）者；

（6）妊娠和哺乳期妇女；

（7）不耐受或拒绝接受针刺/电针者。

4.剔除与脱落标准

（1）出现严重不良事件，根据医生判断停止试验者；

（2）试验过程中病情进展，影响疗效和安全性判定者；

（3）受试者依从性差（试验治疗依从性＜80%），或自动中途接受其它治疗者；

5.中止研究标准

（1）研究过程中出现严重安全性问题；

（2）针刺效果远低于西药组，本次研究不具有临床价值。

六、治疗方案

1.脑卒中基础治疗

针对脑卒中及其高危基础病（如高血压、糖尿病等），两组患者均参考 2019 年版《中国脑血管病临床管理指南》予以对症治疗。

2. 治疗方案

针对脑卒中功能障碍采用按照《针灸学》教材中“中风病”针刺方法进行常规针刺治疗。

取穴：水沟、内关（双侧）、三阴交（患侧）、极泉（患侧）、尺泽（患侧）、委中（患侧）、合谷（患侧）、足三里（患侧）。

操作：患者取仰卧位，75%酒精皮肤消毒，采用一次性针灸针(华佗牌，苏州医疗用品厂有限公司，规格：0.25mm×40mm)进行针刺。水沟向鼻中隔方向斜刺 5～10mm，雀啄手法至眼球湿润为度；内关直刺 10～15mm，提插泻法 1 min；极泉、尺泽、三阴交、委中以患肢抽动 3 次为度，其余穴位针刺深度 20～25mm，留针 30 分钟。每周针刺 6 次，共治疗 4 周。

（1）对照组：口服艾司西酞普兰片 10mg Qd，每日服用，共 4 周。

（2）试验组：增加抗抑郁针刺治疗。

取穴：百会、印堂、风府、耳甲。

操作：患者取半卧位，病床抬高 45°，75%酒精消毒后先进行“醒脑开窍”针刺，方法同前，得气后再针刺抗抑郁相关穴位。其中百会平刺 3～5mm，印堂向鼻根斜刺 10～15mm，风府针向喉结 15～20mm，耳甲相当于耳穴心、肾位置直刺 2～3mm，随后百会、印堂，耳穴心、肾各连一组电针（华佗 SDZ-IIB 型电针仪），频率为 2/15 Hz，强度 2 mA，时间 30min，双侧耳穴交替针刺/电针。每周针刺 6 次，共治疗 4 周。

七、疗效性评价指标

1.观察指标

1.1 人口学资料：年龄、性别、身高、体重、文化程度、病程、合并用药情况等。

1.2 一般体格检查：体温、心率、呼吸、血压等。

2.疗效指标

2.1 主要疗效指标

汉密尔顿抑郁量表（HAMD-17）：用于评定抑郁障碍患者的抑郁症状严重程度，评分内容包括抑郁症的抑郁情绪、有罪感、自杀、入睡困难、睡眠不深、早醒、工作和兴趣、迟缓、激惹、精神性焦虑、躯体性焦虑等 17 个症状条目，目前应用最广。

2.2 次要疗效指标

①临床有效率：以 HAMD-17 评分减少值计算疗效指数并进行疗效判定。

②抑郁自评量表（Self-rating depression scale，SDS）：用于卒中后抑郁患者自我评估，包含 20 个项目，临床上较为常用。

③NIHSS：用于评定脑卒中患者神经功能缺损症状严重程度。

④改良 Barthel 指数(MBI)：用于评估脑卒中患者生活自理能力，评分越高，患者自理能力越强。

⑤实验室指标：血清 IL-1β、IL-10、5-HT、BDNF 水平测定。

⑥血清代谢组学分析。方法：血清样本在室温解冻后，吸取 100 μL 转移到新的试管内，在每个样本中分别添加 300 μL 甲醇和 10 μL 内标（2-氯苯丙氨酸 2.5 g/L）。样品混合并离心后，吸取 200 μL 上层清液至样本管中用于液相色谱串联质谱的检测，检测设备为 Acquity TM UPLC-Q-TOF-MS 平台（Alfa Chemistry，美国）。获得的数据先进数据特征的提取，在 R 中进行 XCMS 处理后，进行归一化处理并形成二维数据矩阵。

2.3 疗效判定标准

根据尼莫地平法以汉密尔顿抑郁量表（HAMD-17 项）减分值计算疗效指数，计算公式为：[（疗前总积分-疗后总积分）/疗前总积分]×100%。疗效判定如下：

痊愈：症候积分减少≥75%；

显效：50%≥症候积分减少＜75%；

有效：25%≥症候积分减少＜50%；

无效：症候积分减少＜25%。

总有效率=（痊愈+显效+有效）/总例数×100%

3.安全性指标

可能出现的不良反应症状、不良反应发生率。

4.观察时点

量表于干预前、2 周、4 周进行评估，血清指标以及代谢组学分析于治疗前和治疗 4 周后检测。

5.统计分析

所有数据均应用 SPSS 26.0 计软件进行统计学分析，计量资料先进行正态性检验及方差齐性检验，其符合正态分布者采用独立样本 t 检验，不符合正态分布者采用非参数检验。计数资料用卡方（χ2）检验。等级资料用非参数检验。以 P＜0.05 为差异有统计学意义。

6.不良事件的记录和报告

6.1 不良事件的记录

在病例报告表中，设置“不良事件记录表”，要求研究者如实填写不良事件的发生时间、严重程度、持续时间、采取的措施和转归。

6.2 不良事件报告

在试验中如出现严重不良事件，研究者必须立即采取措施，保护受试者安全，并及时报告课题研究负责单位和伦理委员会。研究者要在报告上签名并注明日期。申办者将保证满足所有法律法规要求的报告程序。

6.3 严重不良事件的处理

在试验中如出现严重不良事件，如患者出现脑血管意外，心梗等应立即停止本试验，对症予以处理；如患者为初次接受针灸治疗，医者应做好解释工作，以防出现晕针现象。若出现晕针应参考《刺法灸法学》（规划教材）中针刺意外进行处理，立即停止治疗，让患者取头低脚高位平卧，休息片刻，给与饮用温开水；重者在上述处理的基础上，可灸百会，气海、关元、神阙；若仍不能缓解应对症予以紧急治疗。如起针时出现血肿立即按压止血 1 分钟，并嘱患者于当日冷敷，次日可热敷；若出现局部感染，予以抗炎治疗。

八、数据管理

所有与数据收集有关人员都需要经过确认，并描述他们的工作内容。临床研究者必须根据原始资料信息准确、及时、完整、规范地填写 CRF。CRF 数据的修改必须遵照标准操作程序，保留修改痕迹。一旦发现其中有错误或差异，应通知研究者，以确保所有数据的记录和报告正确和完整。必要时可根据研究方案变化对数据管理计划进行及时更新与修订，但需要有相应的手续来完成。数据管理人员应对方案中规定的主要和次要有效性指标、关键的安全性指标进行充分的核查以确保这些数据的正确性和完整性。

九、质量控制

进行实施过程质量控制和数据质量控制。包括研究者实施依从性控制、受试者依从性控制、随访信息完整性控制。数据质量控制包括数据监管人员组成、职责及工作流程、录入过程、期中分析计划、研究终止标准、数据核查、数据清洗等。

十、研究伦理学要求及声明

本临床研究将遵循赫尔辛基宣言（2010 年版）和中国有关临床研究规范、

法规进行。在研究开始前，由课题负责单位伦理委员会批准后，方可实施本研究。每位患者入选本研究前，研究医生有责任以书面文字形式，向其或其指定代表完整的、全面地介绍本研究的目的、程序和可能的风险。应让患者知道他们有权随时退出。入选前必须给每位患者一份知情同意书。研究医生有责任确保每位患者进入研究之前签署知情同意书，并保留在研究档案中。

对有妊娠可能的患者，必须告知患者如在研究期间怀孕，本研究可能对胎儿产生风险，患者需同意在研究中必须采取避孕措施才能参加本研究，如果怀疑患者做不到，则不能让其进入本研究。

因本研究涉及临床试验，研究者声明无论相关研究结果阳性或阴性，均会将相关结果公开发表。

参考文献：参考文献：

[1] Towfighi A, Ovbiagele B, El Husseini N, et al. Poststroke Depression: A Scientific Statement for Healthcare Professionals From the American Heart Association/American Stroke Association[J]. Stroke. 2017;48(2):e30-e43.

[2] 李世明,冯为,崔凤伟,等.中国脑卒中后抑郁患病率 meta 分析[J].实用医学杂志,2021,37(16):2058-2064.

[3] Feng C, Fang M, Liu XY. The neurobiological pathogenesis of poststroke depression[J]. TheScientificWorldJournal. 2014:521349.

[4] Cai W, Stewart R, Mueller C, et al. Poststroke depression and risk of stroke recurrence and mortality: protocol of a meta-analysis and systematic review[J]. BMJ open. 2018;8(12):e026316.

[5] Guo J, Wang J, Sun W, et al. The advances of post-stroke depression: 2021 update[J]. Journal of neurology. 2022;269(3):1236-49.

[6] 齐士魁,高静,余明月,等.脑卒中后抑郁发病机制的研究进展[J].卒中与神经疾病,2022,29(05):483-486.

[7] 唐文静,伍思源,杨晨,等.炎症反应与卒中后抑郁[J].中国组织工程研究,2022,26(08):1278-1285.

[8] Kalbouneh HM, Toubasi AA, Albustanji FH, et al. Safety and Efficacy of SSRIs in Improving Poststroke Recovery: A Systematic Review and Meta-Analysis[J]. Journal of the American Heart Association. 2022;11(13):e025868.

[9] Wijeratne T, Sales C, Wijeratne C. A Narrative Review on the Non-Pharmacologic Interventions in Post-Stroke Depression[J]. Psychology research and behavior management. 2022;15:1689-706.

[10] You Y, Zhang T, Shu S, et al. Wrist-ankle acupuncture and Fluoxetine in the treatment of post-stroke depression: a randomized controlled clinical trial[J]. Journal of traditional Chinese medicine. 2020;40(3):455-60.

[11] Yin XJ, Wang F, Lin GP, et al. Effects of auricular acupressure on depression in stroke patients: A single-blind randomized controlled trial[J]. Complementary therapies in clinical practice. 2022;48:101596.

[12] Li MH, Zhang B, Meng ZH, et al. Effect of Tiaoshen Kaiqiao acupuncture in the treatment of

ischemic post-stroke depression: a randomized controlled trial. Journal of traditional Chinese medicine[J]. 2017;37(2):171-8.
[13] Cai W, Ma W, Li YJ, et al.Efficacy and safety of electroacupuncture for post-stroke depression: a randomized controlled trial[J]. Acupuncture in medicine : journal of the British Medical Acupuncture Society. 2022;40(5):434-42.
[14] 黄海鹏,于斌,管其凡,等.针刺治疗脑卒中后抑郁的临床研究进展[J].中国老年学杂志,2022,42(18):4617-4621.
[15] Zhang L, Chen B, Yao Q,et al. Comparison between acupuncture and antidepressant therapy for the treatment of poststroke depression: Systematic review and meta-analysis[J]. Medicine. 2021;100(22):e25950.
[16] Hang X, Li J, Zhang Y, et al. Efficacy of frequently-used acupuncture methods for specific parts and conventional pharmaceutical interventions in treating post-stroke depression patients: A network meta-analysis[J]. Complementary therapies in clinical practice. 2021;45:101471.
[17] Wang XF, Cai W, Wang YP,et al.Is Electroacupuncture an Effective and Safe Treatment for Poststroke Depression? An Updated Systematic Review and Meta-Analysis[J]. BioMed research international. 2021;2021:8661162.
[18] 蔡娲,高行,魏溪芳,等.针刺治疗卒中后抑郁症的机制研究进展[J].中医药导报,2021,27(09):164-167.
[19] Li M, Ding R, Yang X, Ran D. Study on Biomarkers Related to the Treatment of Post-Stroke Depression and Alternative Medical Treatment Methods. Neuropsychiatr Dis Treat. 2022 Aug 26;18:1861-1873.

十一、附表

1.汉密尔顿抑郁量表（HAMD-17）

汉密尔顿抑郁量表（HAMD-17 项）(请在最接近患者真实情况的那个答案前的“□”里面打“×”)	
1 抑郁情绪	□0 无症状<br>□1 只在问到时才诉述<br>□2 在言语中自发地表达<br>□3 不用言语也可从表情、姿势、声音或欲哭中流露出这种情绪<br>□4 病人的自发语言和非自发语言（表情、动作），几乎完全表现为这种情绪
2 有罪感	□0 无症状<br>□1 责备自己，感到自己已连累他人<br>□2 认为自己犯了罪，或反复思考以往的过失和错误<br>□3 认为目前的疾病，是对自己错误的惩罚，或有罪恶妄想<br>□4 罪恶妄想伴有指责或威胁性幻觉
3 自杀	□0 无症状<br>□1 觉得活着没有意义<br>□2 希望自己已经死去，或常想到与死有关的事<br>□3 消极观念（自杀念头）<br>□4 有严重自杀行为
4 入睡困难	□0 无症状<br>□1 主诉有时有入睡困难，即上床后半小时仍不能入睡

	□2 主诉每晚均有入睡困难
5 睡眠不深	□0 无症状<br>□1 睡眠浅多恶梦<br>□2 半夜（晚上 12 点以前）曾醒来（不包括上厕所）
6 早醒	□0 无症状<br>□1 有早醒，比平时早醒 1 小时，但能重新入睡<br>□2 早醒后无法重新入睡
7 工作和兴趣	□0 无症状<br>□1 提问时才诉述<br>□2 自发地直接或间接表达对活动、工作或学习失去兴趣，如感到没精打采，犹豫不决，不能坚持或需强迫自己去工作或活动<br>□3 病室劳动或娱乐不满 3 小时<br>□4 因目前的疾病而停止工作，住院患者不参加 任何活动或者没有他人帮助便不能完成病室日常事务
8 迟缓：指思维和语言缓慢，注意力难以集中，主动性减退。	□0 无症状<br>□1 精神检查中发现轻度迟缓<br>□2 精神检查中发现明显迟缓<br>□3 精神检查进行困难<br>□4 完全不能回答问题（木僵）
9 激越	□0 无症状<br>□1 检查时表现的有些心神不定<br>□2 明显的心神不定或小动作多<br>□3 不能静坐，检查中曾站立<br>□4 搓手，咬手指，扯头发，咬嘴唇
10 精神性焦虑	□0 无症状<br>□1 问到才时诉述<br>□2 自发地表达<br>□3 表情和言谈流露明显忧虑<br>□4 明显惊恐
11 躯体性焦虑：<br>指焦虑的生理症状，包括口干、腹胀、腹泻、打呃、腹绞痛、心悸、 头痛、过度换气和叹息、以及尿频和出汗等	□0 无症状<br>□1 轻度<br>□2 中度，有肯定的上述症状<br>□3 重度，上述症状严重，影响生活或需加处理<br>□4 严重影响生活和活动
12 胃肠道症状	□0 无症状<br>□1 食欲减退，但不需他人鼓励便自行进食<br>□2 进食需他人催促或请求或需要应用泻药或助消化药
13 全身症状	□0 无症状<br>□1 四肢、背部或颈部沉重感，背痛，头痛，肌肉疼痛，全身乏力或疲倦<br>□2 上述症状明显
14 性症状：<br>指性欲减退、月经紊乱等	□0 无症状<br>□1 轻度<br>□2 重度；

	不能肯定，或该项对被评者不适合。（不计入总分）
15 疑病	□$_0$无症状<br>□$_1$对身体过分关注<br>□$_2$反复考虑健康问题<br>□$_3$有疑病妄想<br>□$_4$伴幻觉的疑病妄想
16 体重减轻	□$_0$无症状<br>□$_1$一周内体重减轻 1 斤以上<br>□$_2$一周内体重减轻 2 斤以上
17 自知力	□$_0$知道自己有病，表现为忧郁<br>□$_1$知道自己有病，但归于伙食太差、环境问题、工作过忙、病毒感染或需要休息等<br>□$_2$完全否认有病
注：代码表示分数即 0=0 分，1=1 分，2=2 分，3=3 分，4=4 分	

2.抑郁自评量表（SDS）

**抑郁自评量表（SDS）**<br>填报说明：根据受试者实际情况，在最接近患者真实情况的那个答案前的“□”里面打“×”。	
	评分标准
1 我觉得闷闷不乐，情绪低沉。	□$_1$很少 □$_2$小部分时间 □$_3$相当多的时间 □$_4$ 绝大部分时间
2 我觉得一天之中早晨最好。	□$_4$很少 □$_3$小部分时间 □$_2$相当多的时间 □$_1$ 绝大部分时间
3 我一阵阵哭出来或觉得想哭。	□$_1$很少 □$_2$小部分时间 □$_3$相当多的时间 □$_4$ 绝大部分时间
4 我晚上睡眠不好。	□$_1$很少 □$_2$小部分时间 □$_3$相当多的时间 □$_4$ 绝大部分时间
5 我吃得跟平常一样多。	□$_4$很少 □$_3$小部分时间 □$_2$相当多的时间 □$_1$ 绝大部分时间
6 我与异性密切接触时和以往一样感到愉快。	□$_4$很少 □$_3$小部分时间 □$_2$相当多的时间 □$_1$ 绝大部分时间
7 我发觉我的体重在下降。	□$_1$很少 □$_2$小部分时间 □$_3$相当多的时间 □$_4$ 绝大部分时间
8 我有便秘的苦恼。	□$_1$很少 □$_2$小部分时间 □$_3$相当多的时间 □$_4$ 绝大部分时间
9 我心跳比平时快。	□$_1$很少 □$_2$小部分时间 □$_3$相当多的时间 □$_4$ 绝大部分时间
10 我无缘无故的感到疲乏。	□$_1$很少 □$_2$小部分时间 □$_3$相当多的时间 □$_4$ 绝大部分时间
11 我的头脑跟平常一样清楚。	□$_4$很少 □$_3$小部分时间 □$_2$相当多的时间 □$_1$ 绝大部分时间
12 我觉得经常做的事情并没有困难。	□$_4$很少 □$_3$小部分时间 □$_2$相当多的时间 □$_1$ 绝大部分时间
13 我觉得不安而平静不下来。	□$_1$很少 □$_2$小部分时间 □$_3$相当多的时间 □$_4$ 绝大部分时间
14 我对将来抱有希望。	□$_4$很少 □$_3$小部分时间 □$_2$相当多的时间 □$_1$ 绝大部分时间
15 我比平常容易生气激动。	□$_1$很少 □$_2$小部分时间 □$_3$相当多的时间 □$_4$ 绝大部分时间
16 我觉得作出决定是容易的。	□$_4$很少 □$_3$小部分时间 □$_2$相当多的时间 □$_1$ 绝大部分时间
17 我觉得自己是个有用的人，有人需要我。	□$_4$很少 □$_3$小部分时间 □$_2$相当多的时间 □$_1$ 绝大部分时间
18 我的生活过的很有意思。	□$_4$很少 □$_3$小部分时间 □$_2$相当多的时间 □$_1$ 绝大部分时间
19 我认为如果我死了别人会生活得好些。	□$_1$很少 □$_2$小部分时间 □$_3$相当多的时间 □$_4$ 绝大部分时间
20 平常感兴趣的事我仍然照样感兴趣。	□$_4$很少 □$_3$小部分时间 □$_2$相当多的时间 □$_1$ 绝大部分时间

评分：|__|__|分

3.NIHSS 量表

美国国立卫生院神经功能缺损评分（NIHSS）

填表说明：请使用者仔细阅读每条项目，明确了解各项目的，进行临床查体，然后根据患者的实际情况评分。（由临床医生填写），请在最接近患者真实情况的那个答案前的“□”里面打“×”。

编号	检查	评分标准	得分
1a	意识水平：<br>即使不能全面评价（如气管插管、语言障碍、气管创伤及绷带包扎等），检查者也必须选择1个反应。只在患者对有害刺激无反应时（不是反射）才能记录3分。	0=清醒，反应敏锐<br>1=嗜睡，最小刺激能唤醒病人完成指令、回答问题或有反应<br>2=昏睡或反应迟钝，需要强烈反复刺激或疼痛刺激才能有非固定模式的反应<br>3=仅有反射活动或自发反应，或完全没反应、软瘫、无反应	□$_{0}$=0 分<br>□$_{1}$=1 分<br>□$_{2}$=2 分<br>□$_{3}$=3 分
1b	意识水平提问：<br>月份、年龄。仅对初次回答评分。失语和昏迷者不能理解问题记2分，因气管插管、气管创伤、严重构音障碍、语言障碍或其他任何原因不能完成者（非失语所致）记1分。可书面回答。	0=两项均正确<br>1=一项正确<br>2=两项均不正确	□$_{0}$=0 分<br>□$_{1}$=1 分<br>□$_{2}$=2 分
1c	意识水平指令：<br>睁闭眼；非瘫痪侧握拳松开。仅对最初反应评分，有明确努力但未完成的也给分。若对指令无反应，用动作示意，然后记录评分。对创伤、截肢或其他生理缺陷者，应予适当的指令。	0=两项均正确<br>1=一项正确<br>2=两项均不正确	□$_{0}$=0 分<br>□$_{1}$=1 分<br>□$_{2}$=2 分
2	凝视：<br>只测试水平眼球运动	0=正常<br>1=部分凝视麻痹（单眼或双眼凝视异常，但无被动凝视或完全凝视麻痹）<br>2=被动凝视或完全凝视麻痹（不能被眼头动作克服）	□$_{0}$=0 分<br>□$_{1}$=1 分<br>□$_{2}$=2 分
3	视野:<br>用手指数或视威胁方法检测上、下象限视野	0=无视野缺失<br>1=部分偏盲<br>2=完全偏盲<br>3=双侧偏盲（全盲，包括皮质盲）	□$_{0}$=0 分<br>□$_{1}$=1 分<br>□$_{2}$=2 分<br>□$_{3}$=3 分
4	面瘫	0=正常<br>1=最小（鼻唇沟变平、微笑时不对称）<br>2=部分（下面部完全或几乎完全瘫痪，中枢性瘫）<br>3=完全(单或双侧瘫痪,上下面部缺乏运动,周围性瘫）	□$_{0}$=0 分<br>□$_{1}$=1 分<br>□$_{2}$=2 分<br>□$_{3}$=3 分
5	上肢运动：<br>置肢体于合适的位置：坐位时上肢平举 90°，仰卧时上抬 45°，掌心向下，若上肢在 10 秒内，记 1～4 分。对失语者用语言或动作鼓励，不用有害刺激。依次检查每个肢体，从非瘫痪侧上肢开始。	上肢：<br>0=上肢于要求位置坚持10秒，无下落<br>1=上肢能抬起，但不能维持10秒，下落时不撞击床或其他支持物	□$_{0}$=0 分<br>□$_{1}$=1 分<br>□$_{2}$=2 分<br>□$_{3}$=3 分<br>□$_{4}$=4 分

		2=能对抗一些重力，但上肢不能达到或维持坐位 90° 或卧位 45°，较快下落到床<br>3=不能抗重力，上肢快速下落<br>4=无运动<br>9=截肢或关节融合，解释：<br>5a 左上肢 5b 右上肢	□9=9 分
6	下肢运动：<br>置肢体于合适的位置：下肢卧位抬高30°，若下肢在5秒内下落，记1～4分。对失语者用语言或动作鼓励，不用有害刺激。依次检查每个肢体，从非瘫痪侧上肢开始。	下肢：<br>0=于要求位置坚持5秒，不下落<br>1=在5秒末下落，不撞击床<br>2=5秒内较快下落到床上，但可抗重力<br>3=快速落下，不能抗重力<br>4=无运动<br>9=截肢或关节融合，解释：<br>6a 左下肢 6b 右下肢	□0=0 分<br>□1=1 分<br>□2=2 分<br>□3=3 分<br>□4=4 分<br>□9=9 分
7	共济失调：<br>双侧指鼻、跟膝胫试验，共济失调与无力明显不呈比例时记分。如病人不能理解或肢体瘫痪不记分。	0=无共济失调<br>1=一个肢体有<br>2=两个肢体有，共济失调在：<br>右上肢 1=有，2=无<br>9=截肢或关节融合，解释：<br>左上肢 1=有，2=无<br>9=截肢或关节融合，解释：<br>右上肢 1=有，2=无<br>9=截肢或关节融合，解释：<br>左下肢 1=有，2=无<br>9=截肢或关节融合，解释：<br>右下肢1=有，2=无	□0=0 分<br>□1=1 分<br>□2=2 分<br>□9=9 分
8	感觉：<br>昏睡或失语者可记1或0分。脑干卒中双侧感觉缺失、无反应及四肢瘫痪者、昏迷病人记2分。	0=正常<br>1=轻到中度，患侧针刺感不明显或为钝性或仅有触觉<br>2=严重到完全感觉缺失，面、上肢、下肢无触觉	□0=0 分<br>□1=1 分<br>□2=2 分
9	语言：<br>命名、阅读测试。昏迷病人3分。	0=正常，无失语<br>1=轻到中度：流利程度和理解能力有一些缺损，但表达无明显受限。<br>2=严重失语，交流是通过病人破碎的语言表达，听者须推理、询问、猜测，能交换的信息范围有限，检查者感交流困难。<br>3=哑或完全失语，不能讲或不能理解	□0=0 分<br>□1=1 分<br>□2=2 分<br>□3=3 分
10	构音障碍：<br>若病人气管插管或其他物理障碍不能讲话，记9分。	0=正常<br>1=轻到中度，至少有一些发音不清，虽有困难，但能被理解<br>2=言语不清，不能被理解<br>9=气管插管或其他物理障碍，解释：	□0=0 分<br>□1=1 分<br>□2=2 分<br>□9=9 分
11		0=没有忽视症	□0=0 分

	忽视症：<br>若病人失语，但确实表现为关注双侧，记分正常。	1=视、触、听、空间觉或个人的忽视；或对任何一种感觉的双侧同时刺激消失<br>2=严重的偏身忽视；超过一种形式的偏身忽视；不认识自己的手，只对一侧空间定位	□1=1 分<br>□2=2 分
	注：代码即表示分数。	评分：|__|__|分	

4.改良 Barthel 指数(MBI)

**改良 Barthel 指数(MBI)**（请在备选结果中选择其中之一，并在相应的“□”里划“×”）	
日常活动项目	评分标准
1 进食	□10 完全独立 □8 少量帮助 □5 中等帮助 □2 大量帮助 □0 完全依赖
2 洗澡	□5 完全独立 □4 少量帮助 □3 中等帮助 □1 大量帮助 □0 完全依赖
3 个人卫生	□5 完全独立 □4 少量帮助 □3 中等帮助 □1 大量帮助 □0 完全依赖
4 穿衣（包括系鞋带）	□10 完全独立 □8 少量帮助 □5 中等帮助 □2 大量帮助 □0 完全依赖
5 大便控制	□10 完全独立 □8 少量帮助 □5 中等帮助 □2 大量帮助 □0 完全依赖
6 小便控制	□10 完全独立 □8 少量帮助 □5 中等帮助 □2 大量帮助 □0 完全依赖
7 上厕所（包括便后清理及整理衣服）	□10 完全独立 □8 少量帮助 □5 中等帮助 □2 大量帮助 □0 完全依赖
8 床椅转移	□15 完全独立 □12 少量帮助 □8 中等帮助 □3 大量帮助 □0 完全依赖
9 平地行走（45m）/轮椅操作	□15 完全独立 □12 少量帮助 □8 中等帮助 □3 大量帮助 □0 完全依赖
10 上下楼梯	□10 完全独立 □8 少量帮助 □5 中等帮助 □2 大量帮助 □0 完全依赖
**注：**代码表示分数即 15=15 分 12=12 分 10=10 分 8=8 分 5=5 分 2=2 分 1=1 分 0=0 分	
