# Supplementary material for: Efficacy of acupuncture in the treatment of post-stroke depression: A study protocol of a randomized controlled trial
Source: PLoS One. 2024 Aug 28;19(8):e0303162. doi: 10.1371/journal.pone.0303162 (PMC11357092; doi:10.1371/journal.pone.0303162)
Supplement: Attachment — Submitted filename: Response to Reviewers.docx [file pone.0303162.s004.docx]

Response to Reviewers

Dear reviewers,

We deeply appreciate your review work.

Thank you for raising constructive questions about this study. We have also deeply reflected on and made revisions. The following are responses to the questions raised by two reviewers. If you have any questions, please criticize and contact us.

**Answers to Reviewer 1's Questions:**

Q: Acupoint identification and naming issues remain in sections 2.5.1 and 2.5.2. In 2.5.1: Shuigou (DU26) is said to be needled bilaterally. DU26 has only one location on the midline. Are two needles to be placed? In 2.5.2 use of the acupoint names is inconsistent with Table 2. In the table the Du Mai points are labeled Du 16 and Du 20. In 2.5.2, acupoint names use “GV” rather than DU: “Baihui (GV20)… Fengfu (GV16)” Please change all to “DU” in 2.5.2.

Re: Thank you for your careful guidance. We apologize for these errors and have now standardized the descriptions of acupoints in 2.5.1, 2.5.2 and Table 2, with Baihui being standardized as DU20, Yintang as EX-HN3 and Fengfu as DU16. The standardization of the terminology and names of the locations is very important and will be taken care of in future studies. We will be careful in future studies.

**Answers to Reviewer 2's Questions:**

Q: The author answered the previous question and can accept its correction. Question 2 asked earlier Inclusion criteria 4 Select subjects The HAMD score of 8 to 17 with mild depression. It may affect the difference, resulting in little difference between the two groups, but it will also need to be verified by clinical trials.

Re: Thank you very much for raising this thought-provoking question. In fact, when designing the experiment, we mainly wanted to observe the effect of acupuncture therapy on mild depression after stroke. If we include patients with scores above 17 and severe depression, medication intervention is necessary, and the existing research protocol does not comply with clinical guidelines and ethical requirements. However, the comparison with drug efficacy is not the problem that our research aims to solve. We aim to explore the significance of acupuncture intervention in patients with mild depression after stroke based on the anti-inflammatory mechanism of acupuncture. The comparison of drug efficacy may be the plan for the next step of research. Of course, the question you raised is also a reality, so we have read a lot of related research. We found that a published study also used the same inclusion criteria for HAMD segments[1]. And they also obtained statistically significant results. Moreover, sample size is also a factor that affects the statistical significance of the results. This study used 35 patients in each group, while our study sample consisted of 42 patients in each group. So we believe that this setting is reasonable. If, as you mentioned, the research results do not show significant differences, we will carefully analyze the reasons and discuss them, which also provides an opportunity for future research.

[1] Sun Q, Xu H, Zhang W, Zhou Y, Lv Y. Behavioral Activation Therapy for Subthreshold Depression in Stroke Patients: An Exploratory Randomized Controlled Trial. Neuropsychiatr Dis Treat. 2022 Nov 29;18:2795-2805. doi: 10.2147/NDT.S392403.
